# Frailty phenotype and multi-domain impairments in older patients with chronic kidney disease

**DOI:** 10.1186/s12877-020-01757-8

**Published:** 2020-09-29

**Authors:** Simone Vettoretti, Lara Caldiroli, Giulia Porata, Carlotta Vezza, Matteo Cesari, Piergiorgio Messa

**Affiliations:** 1 Unit of Nephrology, Dialysis and Kidney Transplantation Fondazione IRCCS Ca’ Granda Ospedale Maggiore Policlinico di Milano, Via della Commenda 15, 20122 Milan, Italy; 2 Department of Clinical Sciences and Community Health, Università degli Studi di Milano, Milan, Italy; 3 Unit of Geriatrics Fondazione IRCCS Ca’ Granda Ospedale Maggiore Policlinico di Milano, Milan, Italy

**Keywords:** Frailty phenotype, Comprehensive geriatric assessment, Chronic kidney disease, Malnutrition, Physical performance

## Abstract

**Background:**

Older subjects with chronic kidney disease (CKD) are often affected by multiple geriatric impairments that may benefit from a comprehensive geriatric assessment (CGA). However, ordinary execution of CGA in all these individuals would be unaffordable. We evaluated if Frailty Phenotype (FP) could identify older CKD-patients that may benefit the most from a CGA.

**Methods:**

We evaluated 112 CKD patients not yet on dialysis (age ≥ 65 years, eGFR < 45 ml/min). FP was defined according to the criteria proposed by Fried and co-authors. CGA evaluated four domains (nutrition, physical performance, cognition and depression). Malnutrition was defined in accordance to Malnutrition-Inflammation Score (MIS) and/or by the presence of Protein Energy Wasting syndrome (PEW). Physical performance was evaluated using Short Physical Performance Battery (SPPB) and handgrip strength. Cognitive status was assessed by using Mini Mental State Examination (MMSE) and Clock Drawing Test. Mood was investigated with Geriatric Depression Scale (GDS).

**Results:**

Average age of our cohort was 80 ± 6 years and mean eGFR 24 ± 11 ml/min/1.73 m^2^. Prevalence of frailty was 45%. Frail patients (F-CKD) had higher prevalence of malnutrition (58 vs 29%, *p* = 0.0005), physical impairment (100% vs 78%; *p* < 0.0001), cognitive dysfunction (83% vs 37%; *p* < 0.0001) and depression (50% vs 21%; *p* < 0.001) compared to robust ones (NF-CKD).

Moreover, F-CKD patients had higher probability to have > 2 impaired domains (83% sensitivity and 76% specificity) respect to NF-CKD individuals.

**Conclusions:**

FP is a reliable screening tool to identify older CKD-patients that may benefit from a CGA.

## Background

A growing proportion of patients with chronic kidney disease (CKD) is affected by the peculiar impairments of the geriatric age (i.e. malnutrition, sarcopenia, physical and cognitive impairments [1]). All these conditions worsen the quality of life and the overall prognosis of older patients with CKD. In those patients, the adoption of multi-disciplinary care programs may confer general health and survival benefits [2, 3]. However, the increasing prevalence of older CKD-patients makes the ordinary execution of comprehensive geriatric assessment of all these subjects unaffordable.

Frailty is a clinical construct that describes a state of decline and vulnerability associated with a worse prognosis in terms of quality of life, prevalence of disability and survival [4]. In patients with CKD frailty is a prevalent condition (14–88% depending on study group characteristics and frailty definitions that were used) [5–7] .

Frail CKD-patients are frequently affected by multi-domain impairments [7]. Therefore, the assessment of frailty may represent a reliable tool to individuate older CKD-patients that deserve to be addressed to a comprehensive geriatric assessment since they might benefit the most from a multidisciplinary care program.

Until now there is no gold standard to diagnose frailty in CKD patients [7–9]. Frailty Phenotype (FP) [10] is a clinical tool that is easy to perform in the outpatients setting on a large scale and it has been extensively validated in CKD-patients [7, 11–13] .

We evaluated cross-sectionally the association of FP with: malnutrition, physical impairment, cognitive dysfunction and depression in older outpatients with advanced CKD. Moreover, we tested whether FP can be used as a screening tool to individuate CKD-patients to be addressed to a comprehensive geriatric assessment. Therefore, the primary endpoint of the study was to evaluate the sensitivity and specificity of FP to identify those subjects that were affected by more than one or two geriatric impairments.

## Methods

### Patients characteristics and study design

We evaluated 112 patients attending our outpatient clinic for advanced CKD between 9/2016 and 3/2018 (belonging to the population included in the PROVE study that has already been described elsewhere [14]). We asked to participate to the study to all eligible patients that attended the clinic during the enrollment period, when they came for a control visit. The study had a cross sectional design.

Patients were selected according to the following criteria: age ≥ 65 years old, CKD stages 3b to 5 not yet on renal replacement therapy and relatively stable eGFR over the previous 6 months (with less than 2 ml/min/1.73m^2^ of variation). In order to exclude patients that were unable to fulfill the tasks of the study protocol or whose impairment was mostly determined by the severity of a single concomitant disease, we applied some exclusion criteria that have been reported elsewhere [14]. We determined estimated glomerular filtration rate (eGFR) by CKD-EPI formula [15, 16].

Medical, biochemical and anthropometrical evaluations were collected in a unique visit that was performed in the morning after an overnight fast of at least 12 h.

All patients signed an informed consent to participate to the study (see above “Ethics approval and consent to participate”).

### Assessment of frailty

Frailty was assessed by using the Frailty Phenotype (FP) as it was originally proposed by Fried and co-workers [10]. Frailty was defined by investigating the following items: unintentional weight loss, exhaustion, weakness, slow gait speed and low physical activity. Patients with three or more deranged items have been classified as frail.

In our cohort, we defined the domains of frailty as follows: 1) unwanted weight loss (≥ 4.5 kg of body mass in 12 months); 2) exhaustion (tired ≥4 days per week for more than 3 months); 3) weakness (handgrip strength < 16 kg in females and < 27 kg in males); 4) slowness (4 m course gait test speed > 0.8 m/sec); reduced physical activity (a score < 7 at physical activity scale, that is extensively described below in the physical performance section).

### Nutritional intake, body composition and nutritional status

Daily dietary protein intake was estimated by assessing normalized protein catabolic rate (nPCR). In order to guarantee the accuracy of 24 h urinary collection all patients were given written instructions [17]. Furthermore, we measured 24 h creatinine excretion on the same urine sample. Low protein diet was defined as a nPCR of 0,6–0,8 g/24 h.

We measured: body weight, height, body mass index (BMI, calculated according to Quetelet Index (kg/m^2^)) and mid-arm muscle circumference (MAMC) of the dominant arm [14].

Body composition was analyzed with a multifrequency bioelectrical impedance analysis device (BCM, Fresenius Medical Care, Bad Homburg, Germany). We estimated: lean tissue (LT), fat tissue (FT) and over-hydration (OH). For technical reasons data regarding body composition are available only for 84 patients.

Malnutrition was defined in accordance to the presence of Protein Energy Wasting syndrome (PEW) and/or by individual Malnutrition-Inflammation Score (MIS).

PEW was defined according to the criteria indicated by the International Society of Renal Nutrition and Metabolism [18]. Malnutrition was defined as MIS > 7 as it was previously reported in patients with CKD [19].

### Physical performance

Short physical performance battery (SPPB) evaluates: the capability of maintaining standing balance, walking speed and leg strength by five repetitive chair-stands [20]. Physical performance was considered impaired when SPPB score was < 10 [21].

Handgrip strength was measured with Jamar dynamometer (Sammons Preston Inc., Bolingbrook, IL). Impaired handgrip strength was defined by the following thresholds: < 16 kg in females and < 27 kg in males [10].

The ability to execute Instrumental Activities of Daily Life (IADL) was assessed by using Lawton and Brody’s scale with normal scoring ranges of 0–8 in women and 0–5 in men [22].

Physical Activity Scale (PAS) is designed to assess the degree of physical activity of CKD patients aged ≥65 years and a score < 7 corresponds to reduced overall physical activity [14]. PAS < 7 was used to define the reduction of physical activity in the assessment of FP score.

### Assessment of cognitive impairment and depression

Cognitive status was assessed by using Mini Mental State Examination (MMSE) and Clock Drawing Test.

Mini Mental State Examination (MMSE) is an 11-question measure that tests five areas of cognitive function: orientation, registration, attention and calculation, recall, and language. The maximum score is 30. A score ≤ 23 is indicative of cognitive impairment [23].

Clock Drawing Test is used for screening for cognitive impairment and dementia and as a measure of spatial dysfunction and neglect. The subject is presented with a circular contour and is expected to draw in the numbers on the clock face. Then the subject is asked to draw the hands at a fixed time, 10 min past 11:00). Doing the test requires verbal understanding, memory and spatially coded knowledge in addition to constructive skills [24]. A score < 5 was considered as impaired.

Mood was investigated by using Geriatric Depression Scale (GDS) and a score ≥ 11 was considered indicative of depression [25].

### Biochemical parameters

Biochemical analyses were all executed at the central laboratory of our Institution.

### Domains

**Fig. 1:**
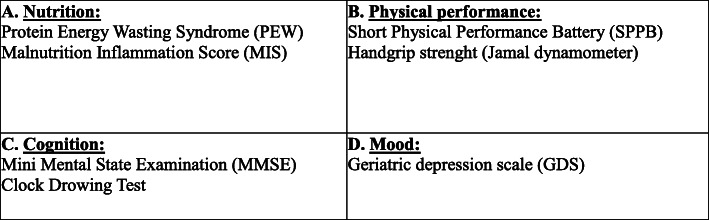
Geriatric Assessment Tools. We adopted a geriatric assessment based on four domains (**a**-**d**). A domain has been judged impaired when at least one of the tools that were used to evaluated it gave a positive result (i.e. the patient had positive PEW test than the nutritional domain was impaired). The cut-offs adopted to define the positivity of the single test are reported in the methods section. Multidomain impairment was defined by altered tests in more than one domain (i.e. having an impairment in 2 domains meant having a positive test in A plus B)

In order to quantify individual geriatric impairment, we considered four domains: nutritional status, physical performance, cognitive function and depression (Fig. 1).

Malnutrition was defined by the presence of PEW and/or by a MIS > 7.

Impaired physical performance was defined by reduced handgrip strength and/or SPPB score < 10.

Cognitive impairment was defined by reduced MMSE score and/or pathological clock test.

Depression was defined by GDS score ≥ 11.

The primary endpoint was to evaluate the sensitivity and specificity of FP to identify those subjects that were affected by > 1 or > 2 impaired domains.

### Statistical analysis

All data are expressed as mean ± SD or median ± IQR as appropriate. Comparison of normally distributed variables was done using Student’s t-test while the comparison of not normally distributed ones was done using the Mann-Whitney “U” test. Proportions and categorical variables were compared using the independent chi-squared (χ2) test or the Fisher’s exact test. We determined: sensitivity, specificity, positive and negative predictive value of FP to identify patients with > 1 or > 2 impaired domains. In order to estimate the strength of the association between FP and multi-domain impairments we calculated the Odds Ratios of these associations. Statistical analysis was carried out with Statview software version 5.0.1.

## Results

### Baseline characteristics of the sample

Patients’ general characteristics are reported in Table 1. Mean age was 80 ± 6, 70% were male, 56% had diabetes, 55% had previous cardiovascular (CV) events. Prevalence of frailty was 50/112 (45%). Of note, although the majority of NF-CKD were males, F-CKD patients were equally distributed between the two sexes.

**Table 1:** Cohort Characteristics

	Overall cohort ***n*** = 112	NF-CKD ***n*** = 62 (55%)	F-CKD ***n*** = 50 (45%)	***P***
Age, yrs	80 ± 6	79 ± 6	81 ± 6	0.08
Males, %	70	87	48	< 0.001
Diabetes, %	56	58	54	0.67
Previous cardiovascular events, %	55	52	60	0.38
eGFR, ml/min/1,73 m^2^	24 ± 11	25 ± 11	24 ± 10	0.48
Creatinine clearance, ml/min/1,73 m^2^	27 ± 14	29 ± 14	23 ± 13	0.025
Urea, mg/dl	101 ± 35	99 ± 33	103 ± 37	0.59
Hemoglobin, gr/dl	12.3 ± 1.3	12.6 ± 1.3	12.0 ± 1.3	0.012

Data are expressed as number (%) or mean ± standard deviation for continuous variables

F-CKD had lower creatinine clearance than NF-CKD (creatinine clearance: 23 ± 13 ml/min/1.73 vs 29 ± 1415 ml/min/1.73m^2^; *p* = 0.025) and lower hemoglobin levels (hemoglobin: 12.0 ± 1.3 g/dl vs 12.6 ± 1.3 g/dl; *p* = 0.012).

### Domains evaluation

#### Nutritional parameters, inflammation and body composition

Nutritional parameters are shown in Table 2. F-CKD patients had lower albumin and prealbumin levels (Albumin 4.0 ± 0.3 g/dl vs 4.1 ± 0.3 g/dl, *p* = 0.04; Prealbumin: 25.8 ± 5.1 mg/dl vs 29.3 ± 5.4 mg/dl, *p* = 0.037), but there were not significant differences in total cholesterol, transferrin, and (25OH) vitamin D levels. Inflammatory status of the two populations was the same as shown by CRP values (CRP: 0.46 ± 0.72 vs 0.46 ± 0.79, *p* = 0.98). The proportion of patients that were prescribed a hypoproteic diet was the same in F-CKD and NF-CKD (35 vs 38% respectively) and estimated protein intake did not differ in the two groups (nPCR: 723 ± 206 vs 787 ± 240 mg/kg/24 h, *p* = 0.13).

**Table 2:** Nutritional Status

	Overall cohort ***n*** = 112	NF-CKD ***n*** = 62 (55%)	F-CKD ***n*** = 50 (45%)	***P***
**Nutritional Parameters**				
Albumin, gr/dl	4.0 ± 0.3	4.1 ± 0.3	4.0 ± 0.3	0.04
Prealbumin, mg/dl	28.3 ± 5.4	29.3 ± 5.4	25.8 ± 5.1	0.037
Total cholesterol, mg/dl	167 ± 37	163 ± 31	171 ± 44	0.28
Transferrin, mg/dl	230 ± 40	229 ± 39	233 ± 41	0.62
Vitamin D (25OH), ng/ml	29 ± 17	30 ± 15	28 ± 19	0.57
CRP, mg/dl	0.46 ± 0.75	0.46 ± 0.79	0.46 ± 0.72	0.98
nPCR, mg/kg/24 h	758 ± 227	787 ± 240	723 ± 206	0.13
Hypoproteic diet, %	37	38	35	0.76
**Body Composition**^a^				
BMI, kg/m2	28.0 ± 4.8	27.7 ± 4.2	28.3 ± 5.5	0.52
MAMC, cm2	25 ± 3	25 ± 3	24 ± 3	0.10
**Bio-impedentiometry results**				
Lean tissue, %	48 ± 12	52 ± 11	43 ± 10	< 0.001
Fat tissue, %	35 ± 9	33 ± 9	39 ± 8	< 0.001
Lean tissue/fat tissue ratio	1.60 ± 0.98	1.84 ± 1.09	1.22 ± 0.61	0.004
Over Hydration, L	1.3 ± 1.7	1.2 ± 1.8	1.5 ± 1.5	0.44
**Malnutrition**				
PEW, %	29	21	38	0.047
MIS > 7%	27	11	46	< 0.001

Data are expressed as mean ± standard deviation for continuous variables

*CRP* C Reactive Protein, *nPCR* Normalized Protein Catabolic Rate, *BMI* Body Mass Index, *MAMC* Mid-Arm Muscle Circumference, *PEW* Protein Energy Wasting, *MIS* Malnutrition Inflammation Score

^a^ data regarding body composition are available only for 84 patients

F-CKD had less lean tissue and more fat than NF-CKD (lean tissue: 43 ± 10 vs 52 ± 11%, *p* < 0.001; fat tissue: 39 ± 8 vs 33 ± 9%, *p* < 0.001), although there were no differences in BMI and MAMC (BMI: 28.3 ± 5.5 vs 27.7 ± 4.2, *p* = 0.52; MAMC: 24 ± 3 cm^2^ vs 25 ± 3 cm^2^, *p* = 0.1).

F-CKD were more malnourished at MIS (46% vs 11%, *p* < 0.001) and had higher prevalence of PEW (38% vs 21%, *p* = 0.047) than NF-CKD (Table 2).

#### Physical performance

F-CKD had worse physical performance than NF-CKD, as demonstrated by lower average scores at SPPB and by reduced handgrip strength and PAS (Table 3). Furthermore F-CKD had higher prevalence of: impaired SPPB (100% vs 53%, *p* < 0.001), reduced handgrip strength (90% vs 58%, *p* < 0.001) and reduced PAS (70% vs 35%, *p* < 0.001). Of note, IADL score showed no differences between the F-CKD and NF-CKD (Table 3).

**Table 3:** Physical Performance

	Overall cohort ***n*** = 112	NF-CKD ***n*** = 62 (55%)	F-CKD ***n*** = 50 (45%)	***P***
**Physical Functions**				
SPPB score	7.5 ± 2.8	9.3 ± 1.8	5.2 ± 2.2	< 0.001
Handgrip strength	21.2 ± 7.5	24.8 ± 6.8	16.8 ± 5.5	< 0.001
IADL score	5.0 ± 1.6	5.0 ± 1.2	4.9 ± 2.0	0.718
Physical activity scale	7.0 ± 4.0	8.8 ± 3.6	5.0 ± 3.6	< 0.001
Impaired SPPB, %	74	53	100	< 0.001
Impaired handgrip strength, %	72	58	90	< 0.001

Data are expressed as number (%) or mean ± standard deviation for continuous variables

*SPPB* Short Physical Performance Battery, *IADL* Instrumental Activities of Daily Life

#### Cognitive and mood evaluation

**Fig. 2:**
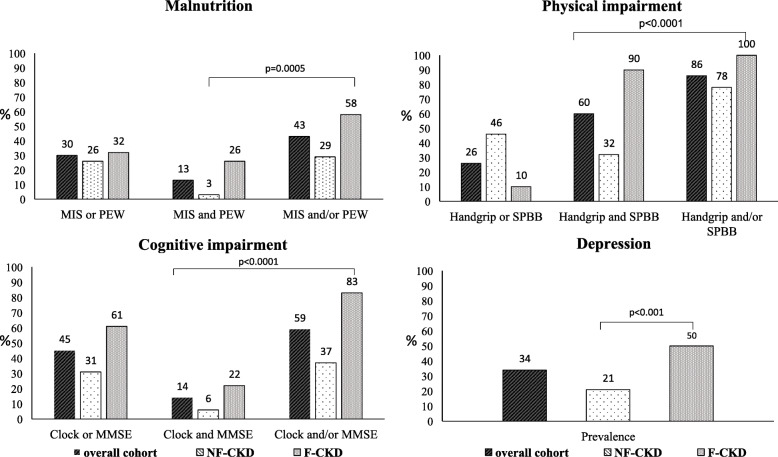
Prevalence of Impaired Indicators and Domain Impairment. In this figure we reported the prevalence of single or associated impairments of the indicators constituting every domain and the prevalence of impaired domains in the overall cohort, as well as in NF-CKD and F-CKD. p refers to the significance between Not Frail-CKD patients (NF-CKD) and Frail-CKD patients (F-CKD)

The two groups showed a significant difference in cognitive evaluation (Table 4). F-CKD patients had worse MMSE (25.3 ± 3.7 vs 27.3 ± 2.4; *p* < 0,001) and Clock test score (2.3 ± 1.9 vs 4.0 ± 1.7; *p* < 0,001) than NF-CKD individuals. Twenty-two percent of F-CKD had impaired MMSE and 83% had an impaired clock test vs respectively 6 and 35% of NF-CKD (MMSE: *p* = 0.016; Clock test: *p* < 0.001). F-CKD had higher GDS score (11.4 ± 5.7 vs 7.5 ± 5.8; *p* < 0.001, Table 4). Depression was more prevalent in F-CKD than in NF-CKD (50 vs 21%, *p* < 0.001; Fig. 2).

**Table 4:** Cognitive and Mood Evaluation

	Overall cohort ***n*** = 112	NF-CKD ***n*** = 62 (55%)	F-CKD ***n*** = 50 (45%)	***P***
**Cognitive status**				
MMSE	26.4 ± 3.2	27.3 ± 2.4	25.3 ± 3.7	< 0.001
Clock test	3.3 ± 2.0	4.0 ± 1.7	2.3 ± 1.9	< 0.001
Impaired MMSE, %	14	6	22	0.016
Impaired Clock test, %	56	35	83	< 0.001
**Mood status**				
GDS score	9.2 ± 6.1	7.5 ± 5.8	11.4 ± 5.7	< 0.001

#### Frailty and single/multi-domain impairments

**Fig. 3:**
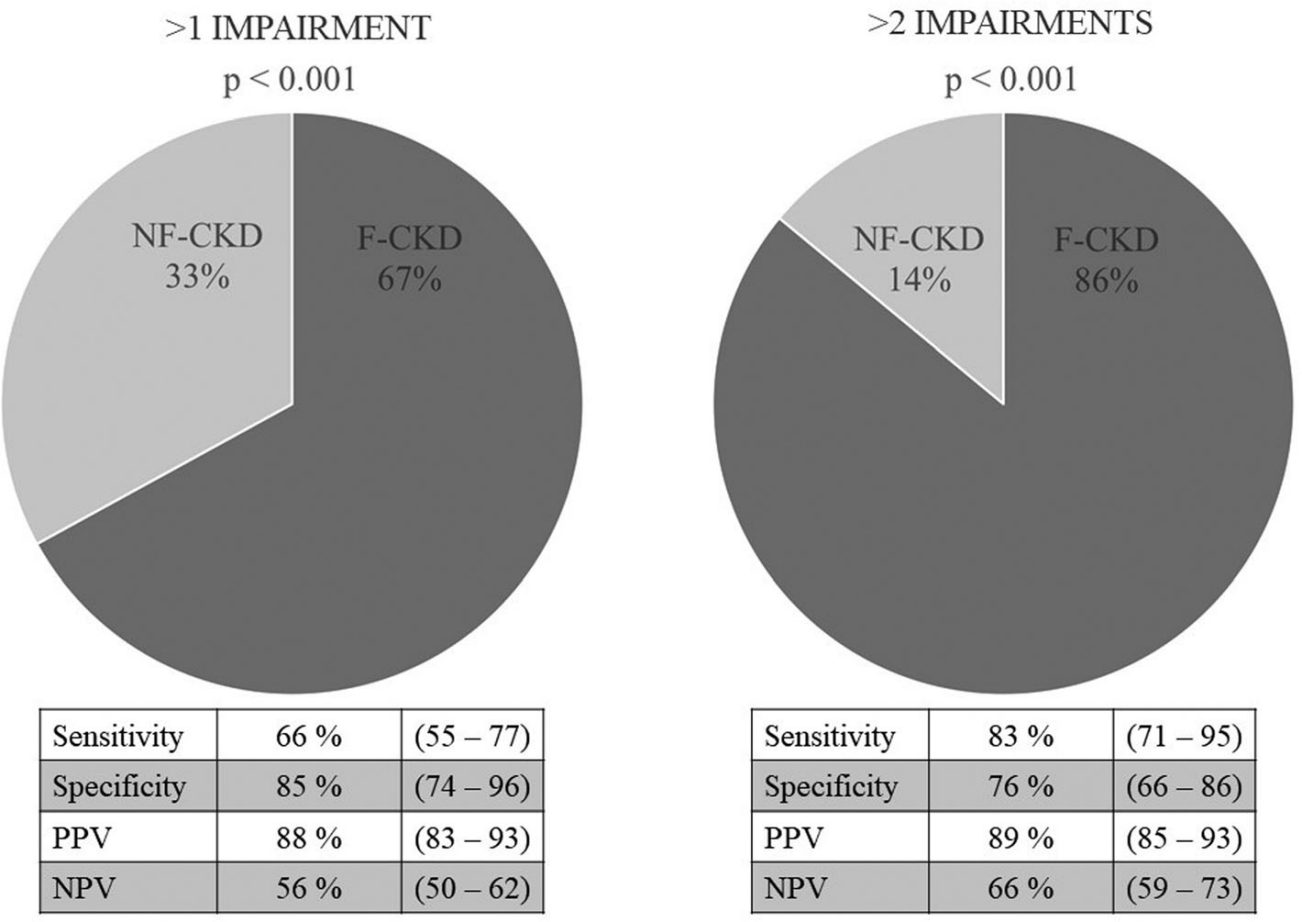
Association Between Frailty and Multi-domain Impairments. Data between () express 95% confidence interval. PPV positive predictive value, NPV negative predictive value

F-CKD patients had higher prevalence of impairments in each of the domains that were considered (Fig. 2). F-CKD patients had also a higher prevalence of multidomain impairments (Fig. 3). Indeed, FP identified CKD-patients with more than one geriatric impairment with a sensitivity of 66% (CI95%: 55–77) and a specificity of 85% (CI95%: 74–96), positive predictive value 88% (CI95%: 83–93), negative predictive value 56% (CI95%: 50–62). Moreover, FP was even more reliable in identifying those patients that had more than two geriatric impairments: sensitivity of 83% (CI95%: 71–95), specificity 76% (CI95%: 66–86), positive predictive value 89% (CI95%: 85–93), negative predictive value 66% (CI95%: 59–73). Overall F-CKD patients were more likely to have more than one (OR 9.51; CI95%: 3.53–25.58) ore more than two impaired domains (15.25; CI95%: 5.72–40.65).

## Discussion

Among older CKD-patients FP has a prevalence of 45% and it is associated with higher prevalence of malnutrition, reduced physical performance, cognitive and mood impairments. Our results suggest also that FP may be used to identify older CKD-patients that are affected by multiple geriatric impairments and that may therefore benefit the most of a comprehensive geriatric assessment.

In previous studies performed in CKD populations prevalence of frailty ranged from 16 to 88% depending on the methods and the definitions that were adopted [7, 11–13, 26–29]. This variability depends on the fact that the definitions used to identify frailty are pretty variegated and take in account different clinical and functional aspects.

Van Loon and co-authors compared the performances of several tools designed to assess the presence of frailty in a population of older CKD-patients that were incident on dialysis [7]. They concluded that all these tools lacked of the discriminating abilities that were necessary to rule out frailty (defined as > 1 geriatric impairment) when they were compared with comprehensive geriatric assessment. Although this study was conducted in patients that were starting the renal replacement therapy while ours considered patients with better renal function (i.e. eGFR: 24 ± 11 ml/min/1.73 m^2^), the results of the two studies are comparable. Both studies found that the prevalence of FP was almost 50% and they showed that FP has a specificity of 85% to exclude patients with less than two geriatric impairment. Additionally, our data demonstrate also that sensitivity and specificity of FP to individuate patients with multiple domain impairments increases in those subjects that have more than two impairments.

Although comprehensive geriatric assessment remains the best tool to evaluate frailty in older CKD- patients, it cannot be ordinarily monitored in all outpatients attending nephrology clinics since it is quite time consuming and it needs specific geriatric competences. Therefore, we suggest that comprehensive geriatric assessment should be performed only in selected individuals that have been identified as frail. Our results suggest that, beyond being an index of vulnerability, FP is a reliable screening tool to identify older CKD-patients that might benefit the most from an integrated and multidisciplinary program of care. Indeed, frailty is not a fixed or inescapable progressive condition, but there are potentially reversible contributors (i.e. poor nutritional status, low mood and physical inactivity) that can be modified by multidisciplinary interventions aimed to improve patients’ outcomes [30].

Our results indicate that frailty is equally prevalent in patients with advanced CKD as well as in those that are incident on dialysis. Therefore, we believe that FP should be systematically assessed in older CKD-patients, since early stages of disease, in order to early identify those that could benefit the most of a comprehensive geriatric assessment. FP is a widely validated method that is focused on physical and subjective aspects [10]. It is based on a pre-defined set of five criteria exploring the presence/absence of signs or symptoms that can easily be assessed in the outpatients setting and it does not take more than 10 min to be concluded.

Notably, although we excluded by our study all patients with previous diagnosis of depression, we found that one third of the overall cohort was actually affected by depression of clinical relevance. Since the prevalence of depression was more than doubled in F-CKD respect to NF-CKD individuals, we suggest that FP might be a reliable screening test to identify those patients that are likely to suffer of misdiagnosed depression.

Our study has some limitations. It is relatively small, even though the number of patients that were enrolled reflects the average of the other studies that were previously performed on the same topic. Furthermore, we adopted quite restrictive inclusion criteria therefore we may have excluded sickest and frailer individuals. However, by excluding those subjects whose frailty and overall outcomes depend mostly on the severity of a single disease, we aimed to focus on those patients that are frequently overlooked because of an apparently favorable prognosis. Therefore, whether we had considered all older patients attending nephrology outpatients it is possible that the actual prevalence of frailty and geriatric impairments would have been even higher than what we found.

## Conclusions

To the best of our knowledge this is the first study that explores whether FP can be used as a screening tool to stratify the risk of geriatric impairment of CKD-patients in the pre-dialysis setting. Overall, we believe that our results support the clinical use of FP to identify older patients with moderate to severe CKD that may benefit the most of a comprehensive geriatric assessment.

## Data Availability

The datasets analyzed in the current study are not publicly available due to the fact that they belong to a larger dataset that is shared in a research consortium involving the University Sacro Cuore of Piacenza and other Departments of the University of Milan. Therefore, some data are currently under analysis also for other research purposes. The original dataset could be provided by the corresponding author, upon the approval of the other members of the consortium, on reasonable request.

## Abbreviations

CKD: Chronic kidney disease
FP: Fried Phenotype
NYHA: New York Herat Association
CGA: Comprehensive geriatric assessment
NF-CKD: Not frail chronic kidney disease patients
F- CKD: Frail chronic kidney disease patients
SPPB: Short physical performance battery
MAMC: Mid arm muscle area
MMSE: Mini mental state examination
MIS: Malnutrition inflammation score
IADL: Instrumental activities of daily life
PAS: Physical activity scale
PEW: Protein energy wasting syndrome
GDS: Geriatric depression scale
